# Endovascular coiling for cerebral aneurysm: single-center experience in Egypt

**DOI:** 10.1186/s41983-018-0040-0

**Published:** 2018-11-07

**Authors:** Mohamed Khaled Elewa

**Affiliations:** Neurology Department, Ain Shams University Hospitals, 38 El-Abbasia, Cairo, 11566 Egypt

**Keywords:** Subarachnoid hemorrhage, Cerebral aneurysm coiling, Outcome

## Abstract

**Background:**

Endovascular management for cerebral saccular aneurysm has evolved in the last decade with evolution in both equipment and material. Coiling is still the mainstay of cerebral aneurysm endovascular management. In Egypt, practice outcome needs evaluation especially at low-volume centers.

**Purpose:**

To discuss the technical and management outcomes of our first symptomatic and asymptomatic cerebral saccular aneurysm case series treated with simple coiling.

**Patients and methods:**

Clinical, treatment, and outcome variables of consecutive symptomatic and asymptomatic cerebral aneurysm cases treated with simple coiling between January 2011 and June 2016 in one center were analyzed.

**Results:**

In 31 patients, 35 aneurysms were found, 34 aneurysms (97.1%) were treated by endovascular coiling, and only one aneurysm (2.9%) was not fit for endovascular treatment. Total occlusion was achieved in 29 aneurysms (82.9%). Neck remnants were present in 4 aneurysms (11.4%). Partial coiling (incomplete occlusion) was achieved in 1 aneurysm (2.9%). Regarding functional outcome (mRS at discharge), 25 patients had good outcome (mRS = 0, 1, 2, 3) and 6 patients had poor outcome (mRS = 4, 5, 6).

**Conclusion:**

The endovascular coiling could be used as a first-choice option for treatment of saccular cerebral aneurysms at our center despite the low case rate.

## Introduction

After the International Subarachnoid Aneurysm Trial (ISAT) [1], endovascular coiling became the first treatment option in many countries. The ISAT trial showed that coiling was associated with 7.4% absolute risk reduction at the outset and significantly fewer dead and dependent patients at 1 year than did clipping. After 10 years follow-up, although the rates of increased dependency did not differ between both groups, the probability of death or dependency was significantly greater in the clipping group than in the coiling group. Rebleeding was more likely after coiling than after clipping, but the risk was small. The Barrow Ruptured Aneurysm Trial (BRAT) has confirmed the superiority of endovascular coiling over the surgical clipping [2]. The rate of poor outcome after 1 year was significantly lower in patients who underwent endovascular coiling in two recent meta-analyses of all major randomized controlled trials comparing coiling and surgical clipping [3, 4].

Our purpose in the current study is to assess and discuss the management and outcome of our first consecutive symptomatic and asymptomatic cerebral aneurysm cases treated with simple coiling.

## Patients and methods

A retrospective review of patients who had underwent endovascular coiling for symptomatic and asymptomatic saccular cerebral aneurysm between January 2011 and June 2016 in Suez insurance hospital was performed. Thirty-five aneurysms in 31 cases were included in the current study. All patients’ aneurysms were fulfilling the following inclusion criteria: ruptured (presenting with acute subarachnoid hemorrhage) or unruptured either presented with mass effect or accidentally discovered (≥ 5 mm in size in the anterior circulation, posterior circulation aneurysms, cavernous segment ICA aneurysms, and aneurysms in patients with past or family history of SAH). Patients with Hunt and Hess grade V (deep coma, decerebrate posturing, or moribund) were excluded. Written consents were obtained from all patients before the procedures. The study was approved by Ain Shams University, Faculty of Medicine Ethical Committee. All patients underwent clinical assessment together with Hunt and Hess scale (H&H scale), Modified Rankin Scale (mRS) on admission, before discharge and during follow-up, brain CT scan ± CSF analysis, Fisher grading, cerebral CT angiography (CTA) or MR angiography (MRA) when necessary, and six vessel digital subtraction cerebral angiography (DSCA) followed by endovascular aneurysmal coiling. Clinical and radiological (3D MRA time of flight or DSCA) follow-up was done 6 months after discharge and then yearly till 5 years. A “good” outcome was defined as a mRS score of 0–2 at discharge; a “poor” outcome was defined as a mRS score of 3–6 at discharge.

## Technique of intervention and periprocedural management

**Fig. 1:**
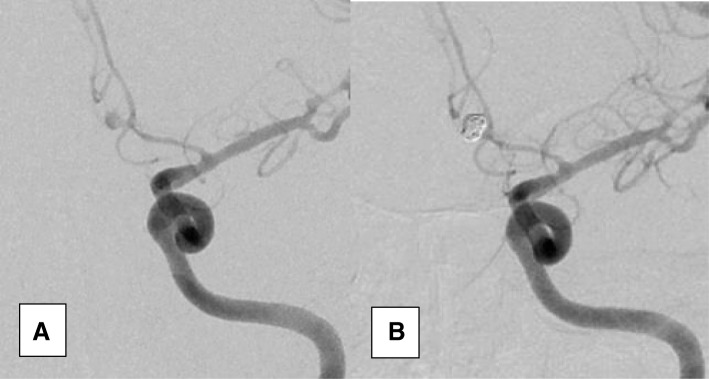
A2 aneurysm before (**a**) and after (**b**) coiling showing complete obliteration (class I according to modified Raymond–Roy Classification). An associated vasospasm is noticed (before intra-arterial nimodipine injection)

All procedures were carried out via femoral puncture. Six vessels DSCA was done for all patients. Once saccular cerebral aneurysm was detected, complete assessment of its angiographic features was done using three-dimension angiography or two-dimension angiography in different projections. Our angiographic assessment aimed to determine the aneurysm site, size, neck type (narrow vs wide neck), presence of vasospasm, presence of daughter aneurysm arising from the main sac, and presence of branch arising from the aneurysm. Narrow neck aneurysm was defined as a dome to neck ratio ˃ 2:1. If multiple aneurysms were found, the bleeding one was treated first. Bleeding aneurysm was identified via the distribution of the blood in the CT scan, the distribution of the vasospasm, aneurysmal shape (lobulated aneurysm or presence of daughter aneurysm), and aneurysmal size. Systemic heparinization was done once the endovascular therapy was decided. Then, selective catheterization of the target vessel (either the internal carotid or the vertebral artery) was done using 6F guiding catheter. Using coaxial system, the pathway to the aneurysm was navigated and then the aneurysmal sac was selectively catheterized using microcatheter. The aneurysmal sac was packed completely as possible with bare platinum detachable coils. The immediate angiographic result was classified via Modified Raymond–Roy Classification (MRRC) into; class I (complete obliteration), class II (residual neck), class IIIa (residual aneurysm with contrast within coil interstices), and class IIIb (residual aneurysm with contrast along aneurysm wall) [5]. Successful procedural outcome was defined as the target aneurysm having complete angiographic occlusion or residual neck according to MRRC in angiographic appearances. In case of severe vasospasm local intra-arterial injection of vasodilator medication (nimodipine or nitroglycerine) was done. If vasospasm did not respond to local injection of vasodilators, balloon angioplasty was used. During follow-up, recurrence was defined as recanalization of a volume within the aneurysm large enough to permit retreatment with endovascular means [6](Fig. 1).

Data were analyzed using Statistical Program for Social Science (SPSS) version 20.0. (SPSS, Chicago, IL) Quantitative data were expressed as mean ± standard deviation (SD). Qualitative data were expressed as frequency and percentage. The following tests were done: independent sample *t* test of significance was used when comparing between two means, chi-square (× 2) test of significance was used to compare proportions between two qualitative parameters, and binary logistic regression was used to predict the outcome of categorical variable based on one or more predictor variables. In all tests, *P* value < 0.05 was considered significant, *P* value < 0.001 was considered highly significant, and *P* value > 0.05 was considered insignificant.

## Results

Patients were assigned to treatment between January 2011 and June 2016. Males and females were almost equally distributed. The mean age was 45.7 ± 13 years (range, 18–71 years), and hypertension was the most prevalent risk factor 13 patients 41.9%. Most of the patients were symptomatic 30 patients (96.8%) either presented with subarachnoid hemorrhage 28 patients (90.3%) or mass effect of 2 patients (6.5%). Only one patient was asymptomatic. According to Hunt and Hess scale, 10 patients (32.2%) were at grades 1 and 2, and on the other hand 21 patients (67.8%) were at grades 3 and 4. According to Fisher grading, 22 patients (71%) were at grades 1, 2, and 9 patients (29%) were at grades 3 and 4. Five patients (16.1%) had vasospasm-related infarction, and five patients (16.1%) developed hydrocephalus necessitating shunt (Table 1). Thirty-five aneurysms were found among 31 patients. Their angiographic features were described in (Table 2).

**Table 1:** Patients characteristics and initial clinical evaluation

Patients characteristics	No.	%
Sex		
Male	15	48.4
Female	16	51.6
Age per years [range (mean ± SD)]	18–71 [45.7 ± 13]	
Family history of subarachnoid hemorrhage	3	9.7
Smoking	12	38.7
Hypertension	13	41.9
Diabetes mellitus type II	4	12.9
Ischemic heart disease	8	25.8
Myocardial infarction	1	3.2
Chronic liver disease	2	6.5
Patients with bleeding aneurysm	28	90.3
Patients with aneurysm causing mass effect	2	6.5
Patients with asymptomatic aneurysm	1	3.2
Hunt and Hess scale		
Grades 1–2	24	45.2
Grades 3–4	17	54.8
Cranial nerve palsy	4	12.9
Modified Rankin Scale		
Scores 0–2	10	32.2
Scores 3–5	21	67.8
Fisher grading		
Grades 1–2	22	71
Grades 3–4	9	29
Vasospasm-related infarction	5	16.1
Hydrocephalus necessitating shunt	5	16.1

**Table 2:** Angiographic assessment

	No. (*N* = 35)	%
Site		
A Comm	12	34.3
A1–A2	3	8.6
P Comm	7	20.0
MCA	4	11.4
Ophthalmic	3	8.6
ICA bifurcation	2	5.7
Pericallosal	1	2.9
Cavernous	1	2.9
Basilar apex	1	2.9
Proximal PICA	1	2.9
Size		
< 5 mm	17	48.6
> 5 and < 10	17	48.6
> 10	1	2.9
Neck type		
Narrow	23	65.7
Wide	12	34.3
Branch arising from the aneurysm	5	14.3
Vasospasm	13	37.1

*A Comm* anterior communicating artery, *A1* A1 segment of anterior cerebral artery, *P Comm* posterior communicating artery, *MCA* middle cerebral artery, *ICA* internal carotid artery, *PICA* posterior inferior cerebellar artery

**Fig. 2:**
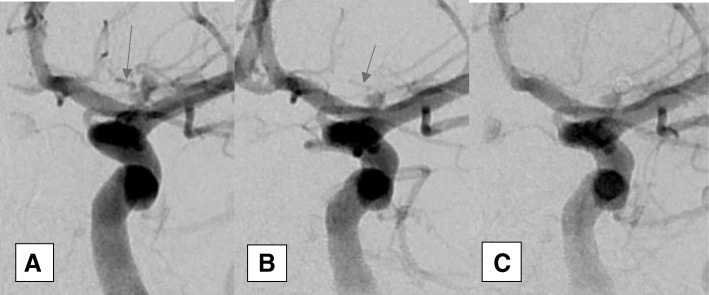
Carotid terminus aneurysm before (**a**) and after coiling (**b**) and (**c**) showing residual neck (class II) according to modified Raymond–Roy Classification. The aneurysmal neck was left intentionally for preservation of small branch that was seen arising from it (arrows)

**Fig. 3:**
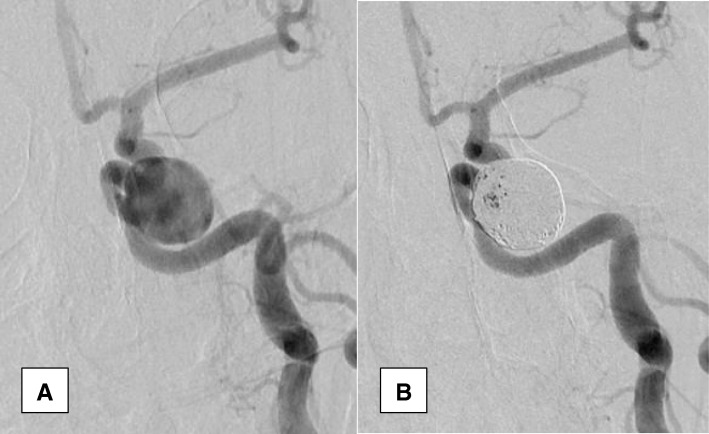
Carotid cavernous aneurysm before (**a**) and after (**b**) coiling. Residual aneurysm with contrast within coil interstices is noticed (class IIIa according to modified Raymond–Roy Classification)

Among 31 patients, 34 aneurysms were treated by endovascular coiling without remodeling techniques out of 35 aneurysms. The untreated aneurysm belongs to young adult patient. She was presented with bleeding left ICA bifurcation aneurysm associated with vasospasm which was treated by endovascular means. She had another asymptomatic tiny (1 × 1 × 1.5) anterior communicating artery aneurysm which was not fit for endovascular treatment. Complete obliteration was achieved in 29 aneurysms (82.9%) (Fig. 1). Residual neck was present in 4 aneurysms (11.4%) (Fig. 2). Residual aneurysm with contrast within coil interstices was found in 1 aneurysm (2.9%) (Figs. 2 and 3). Regarding functional outcome (mRS at discharge); 23 patients had good outcome (mRS = 0, 1, 2) and 8 patients had poor outcome (mRS = 3, 4, 5, 6). Two patients died post operatively before discharge one due to chest infection and the other due to pulmonary embolism (Table 3). Six patients discharged with significant neurological deficit (5 due to vasospasm related infarction and one due to parenchymal hematoma). In five aneurysms, we had branches which originated from the aneurysmal neck, and we succeeded to treat these aneurysms without ischemic complication. The mean of the follow-up duration was 33.03 ± 15.96 months (the range was from 6 to 60 months) for 29 patients. Permanent complete obliteration was achieved in 23 patients (79.3%). Residual neck was found in 3 patients (10.3%). Residual aneurysm was found in 3 patients (10.3%) (Fig. 3). None of our patients developed delayed ischemia or rebleeding. Significant aneurysmal recurrence occurred in 2 cases which required retreatment. Retreatment was not associated with adverse events (Table 4).

**Table 3:** Procedural outcome

	No.	%
Treated aneurysms	34	97.1
Aneurysm not fit for endovascular treatment	1	2.9
MRRC (immediate angiographic result)		
I (complete obliteration)	29	82.9
II (residual neck)	4	11.4
IIIa (residual aneurysm with contrast within coil interstices)	1	2.9
IIIb (residual aneurysm with contrast along aneurysm wall)	0	0.0
mRS at discharge		
0	15	48.4
1	6	19.4
2	2	6.5
3	2	6.5
4	3	9.7
5	1	3.2
6	2 (died)	6.5
Procedural-related mortality	0	0.0
Non-procedural related mortality	2	5.7
Hydrocephalus necessitating shunt	2	5.7

*MRRC* Modified Raymond–Roy Classification

**Table 4:** Long-term follow-up

	No.	%
Duration of follow-up (m)		
Range [mean ± SD]	6–60 [33.03 ± 15.96] months	
Delayed ischemia	0/29	0.0
MRRC		
I	23/29	79.3
II	3/29	10.3
IIIa and IIIb	3/29	10.3
Rebleeding	0/29	0.0
Retreatment (coiling)	2/29	6.9

*MRRC* Modified Raymond–Roy Classification

A comparison between patients with poor outcome and patients with good outcome was done (univariate analysis). The comparison included clinical, radiological, angiographic, and procedural variables. The presence of history of myocardial infarction was found to be significantly higher (*P* = 0.038) in those with poor outcome. Hunt and Hess scale, mRS and Fisher grading were significantly different between the two groups (*P* = 0.023), (*P* = 0.044), and (*P* = 0.006), respectively. The presence of vasospasm was significantly higher (*P* = 0.022) in the poor outcome patients. On the other hand, the presence of vasospasm-related infarction was a highly significant difference between the two groups (being more in poor outcome group) (*P* < 0.001). There was no difference between the two groups according to procedural variables (immediate angiographic results) (Table 5).

**Table 5:** Comparison between patients with poor outcome and patients with good outcome according to variables that showed significant differences

Patients characteristics	Poor outcome patients (*n* = 6)		Good outcome patients (*n* = 25)		× 2/*t*	*P* value
	No.	%	No.	%		
Myocardial infarction	1	16.7	0	0.0	4.306	0.038
Hunt and Hess scale						
1–2	0	0.0	14	56.0	6.075	0.023
3–4	6	100.0	11	44.0		
Admission mRS						
0–2	0	0	10	40.0	3.963	0.044
3–5	6	100	15	60		
Fisher grading						
1–2	1	16.7	21	84.0	7.630	0.006
3–4	5	83.3	4	16.0		
Vasospasm-related infarction	4	66.7	1	4.0	14.047	< 0.001
Vasospasm	5	83.3	8	32.0	5.236	0.022
Hydrocephalus necessitating shunt	2	33.3	0	0.0	8.908	0.003

*mRS* Modified Rankin Scale

Multivariate analysis showed that Hunt and Hess scale, admission mRS, Fisher grading, vasospasm-related infarction, and hydrocephalus necessitating shunt had a significant association with poor outcome. On the other hand, the presence of history of myocardial infarction or presence of vasospasm was not association with the outcome (Table 6).

**Table 6:** Logistic regression analysis for variables which are possibly affecting the outcome

Factors	*B*	Sig.	Exp(B)	C.I. 95%	
				Lower	Upper
MI*	0.187	0.243	0.928	0.696	1.253
Hunt and Hess scale	0.570	0.018	1.100	0.492	0.886
Admission mRS	0.182	0.039	1.166	0.700	1.260
Fisher grading	0.166	0.020	0.946	0.710	1.277
Vasospasm-related infarction	0.206	0.017	0.912	0.684	1.231
Vasospasm	0.309	0.959	0.656	0.825	1.485
Hydrocephalus necessitating shunt	0.748	0.047	0.833	0.875	1.574

*MI* myocardial infarction, *mRS* Modified Rankin Scale

## Discussion

The preferable treatment of cerebral aneurysms at our center is endovascular coiling. The ISAT showed that for bleeding aneurysms suitable for both techniques, endovascular coiling should be the first-choice treatment [1]. The ISAT randomized only aneurysms considered treatable by both techniques, resulted in only 22% of patients presented with bleeding aneurysm being included in the study. This point generates criticism due to selection bias [7]. Another point that should be considered in the ISAT that endovascular coiling was done by highly experienced radiologists, and therefore, results cannot be generalized to other operators [8]. Other limitations of the ISAT were the lack of patients with poor clinical condition (94% of the patients had World Federation of Neurosurgical Societies grades 1–3) and the limited number of patients with posterior circulation aneurysms (more than 90% patients had small anterior circulation aneurysms). The clarity study was the first multicenter, and prospective registry aimed to evaluate endovascular coiling as the first-choice treatment in consecutive patients with ruptured aneurysms treated by non-selected operators. The study avoided the methodological limitations of the ISAT. Nineteen French centers participate in the study with 34 operators. The study included 405 patients treated with GDC coils. The clarity study showed that the results of coiling as a first choice in non-selected bleeding aneurysms by non-selected operators were like those of the ISAT [7]. Also, the BRAT investigators tried to overcome ISAT concerns in their study. The BRAT assigned every SAH patient randomly in an alternate fashion to either clipping or coiling (regardless suitability for treatment modality). The BRAT study confirmed the ISAT results. After 1 year, the outcome of coil embolization was better than surgical clipping. The rate of patients with poor outcome (mRS > 2) was 23.2% in endovascular group versus 33.7% in the surgical group (*P* = .02, intention-to-treat analysis) [9]. In a meta-analysis of published trials comparing endovascular coiling versus surgical clipping, the odds of poor outcome were higher in surgical group, despite the higher risk of early rebleeding after coiling [3].

Coiling with bare platinum coils remains the mainstays of endovascular treatment. Despite the evolution of coil coating technologies to encourage aneurysmal occlusion, the results were disappointing. Randomized controlled trials testing Cerecyte coils versus bare platinum coils and hydrogel-coated coils versus bare platinum showed no additional benefit over bare platinum coils regarding primary outcome (recurrence was lower in hydrogel coil group) [10, 11]. Piotin and his colleagues found that polyglycolic-lactic acid (PGLA)-coated coils (Matrix coils) did not show better recanalization rates than bare platinum coils during long-term follow-up [7, 12]. Angiographic outcome of cerebral aneurysms treated by endovascular coiling was not affected by approved coil type used [13]. All patients in the current study were treated with bare platinum coils.

The immediate angiographic outcome showed complete obliteration in 29 aneurysms (82.9%), residual neck in 4 aneurysms (11.4%), and residual aneurysm with contrast within coil interstices in 1 aneurysm (2.9%). The immediate angiographic result of the (AMERICA) study showed that 52/100 aneurysms had complete occlusion, and 33/100 aneurysms had residual neck or dog ear and 15/100 aneurysms residual sac [14]. The Cerecyte coil trial included 500 patients randomized for treatment with either Cerecyte or bare platinum coils for ruptured or unruptured aneurysms. The bare platinum coils arm included 251 patients: 120 patients presented with rupture aneurysms and 131 patients presented with unruptured aneurysms. Seven patients had failed embolization. The image could not be assessed in 10 patients. The immediate angiographic results in the bare platinum arm (231 aneurysms) as reviewed by the core lab was as follows: complete occlusion 59 aneurysms (25.5%), neck remnant 116 aneurysms (50.2%), sac filling or incomplete occlusion 27 aneurysms (11.7%), and coils overlapping the aneurysm neck aneurysms (12.5%) [10]. The better immediate angiographic results in current study could be explained by the small size of the sample and the angio-anatomical characteristics of the studied aneurysm that favors simple coiling over coiling with remodeling techniques. Also, the retrospective data derived from our study are limited by the lack of core-laboratory adjudication of clinical and angiographic outcomes, as the treatment center was responsible for reporting patients’ clinical and angiographic data. In the previous endovascular aneurysm coiling trials, core-laboratory review has revealed lower occlusions results [11, 15].

Favorable outcome (mRS 0, 1, 2) at discharge was achieved in 23 patients (74.3%); on the other hand, unfavorable outcome (mRS 3, 4, 5, 6) occurred in 8 patients (25.7%). The ISAT included 1065 patients in the endovascular arm, 787 patients (73.9%) had favorable outcome (mRS 0, 1, 2), and 278 patients (26.1%) had unfavorable outcome (mRS 3, 4, 5, 6) 2 months following endovascular coiling. After 1 year follow-up for 1063 patients, 813 patients (76·5%) had favorable outcome and 250 patients (23·5%) had unfavorable outcome [16]. The Axium MicroFX for Endovascular Repair of Intra Cranial Aneurysm Study (AMERICA) included 99 patients. They were treated with Polyglycolic/polylactic acid (PGLA)-coated coils for 100 cerebral aneurysms (either ruptured or unruptured). Twenty-two percent of patients were presented with acute aneurysmal subarachnoid hemorrhage (SAH). Forty-eight percent of aneurysms treated simple coiling, other aneurysms treated with assisted coiling. It showed that 86 patients out of 99 patients (86.9%) had favorable outcome (mRS 0, 1, 2) and 13 patients (13.1%) had unfavorable outcome (mRS 3, 4, 5, 6) at discharge. After follow-up, 83 patients out of 89 patients (93%) had favorable outcome (mRS 0, 1, 2) and 6 patients (6.7%) had unfavorable outcome (mRS 3, 4, 5, 6) [14]. Regarding clinical outcome, our results were similar to the ISAT results, even better if we take in consideration our patients population characteristics (90.3% of our patients had ruptured aneurysms). This could validate our policy for choosing endovascular coiling as the first option at our center. The AMERICA study showed better results that can be explained by its patient population characteristics (only 22% of patients underwent treatment after acute aneurysmal subarachnoid hemorrhage).

After 33 months, a mean follow-up duration, permanent total occlusion was achieved in 23 patients (79.3%) out of 29 patients. Significant neck remnants were found in three patients (10.3%). Major aneurysmal recurrence occurred in three patients (10.3%). In the ISAT, after 1 year follow-up for 988 in the endovascular arm, 881 patients (89%) underwent angiogram 584 patients (66%) achieved complete aneurysmal occlusion, 228 patients (26%) had neck remnants, and 69 patients (8%) had incomplete occlusion [16]. The AMERICA study showed that after approximately 5.2 months follow-up out of 85 aneurysms completed follow-up, 60 aneurysms (70.6%) had complete occlusion, 17 aneurysms (19.9%) had residual neck, and 8 aneurysms (9.4%) had residual sac [14]. In the Cerecyte study, 13 patients were not available for the first follow-up (either lost or died). One hundred eighteen out of 218 (54%) treated aneurysm with bare platinum coils had complete occlusion, stable occlusion, or improved occlusion [10]. The better results here can be explained on the same basis as the immediate angiographic results.

None of our patients developed rebleeding during follow-up period. The BRAT reported no rebleeding up to 2 years after coiling [2]. Asymptomatic significant aneurysmal recurrence occurred in 2 cases out of 29 cases completed their follow-up (6.9%). One case after 22 months follow-up and the other after 35 months, they required retreatment (endovascular coiling). Retreatment passed uneventful. Daugherty and his colleagues argue that the decision of retreatment is subjective to some extent [17]. In the Cerecyte study, out of 230 aneurysms treated with bare platinum coils, 8 aneurysms (3.5%) were retreated. Retreatment median time was 6 months with an interquartile range of 7 to 11.5 months (range, 4–34 months). Retreatment was not associated with adverse events [10]. The high incidence of recurrence in the current study may be explained by small size of the sample and the long-time follow-up.

In the current study, univariate and multivariate analysis showed that factors, which were associated with poor outcome (Hunt and Hess scale, admission mRS, fisher grading, vasospasm related infarction, and hydrocephalus necessitating shunt had a significant), were mainly related to severity of the bleeding. None of these factors was related to the technical aspects of the procedure.

## Conclusion

The current results validate the endovascular option as the first-choice option for treatment of saccular cerebral aneurysms at our center despite the low case rate. Several limitations should be considered in the current study as follows: the lack of core-laboratory assessment, the retrospective nature, the potential patient selection for treatment bias, and the small size of the sample.
